# Acquisition of Interleukin-6 Production Ability Over Time With Pheochromocytoma

**DOI:** 10.1210/jcemcr/luad106

**Published:** 2023-09-02

**Authors:** Yohei Toyoda, Ko Aiga, Mitsuhiro Kometani, Takashi Yoneda

**Affiliations:** Department of Internal Medicine, Keiju Medical Centre, Nanao, Ishikawa 926-8605, Japan; Department of Health Promotion and Medicine of Future, Kanazawa University Graduate School of Medicine, Kanazawa, Ishikawa 920-8641, Japan; Department of Health Promotion and Medicine of Future, Kanazawa University Graduate School of Medicine, Kanazawa, Ishikawa 920-8641, Japan; Department of Health Promotion and Medicine of Future, Kanazawa University Graduate School of Medicine, Kanazawa, Ishikawa 920-8641, Japan

**Keywords:** pheochromocytoma, interleukin-6

## Abstract

Pheochromocytoma is a tumor of chromaffin cells causing catecholamines overproduction. Interleukin-6 (IL-6), a cytokine, is central to inflammation and immunity. Few studies have reported IL-6–producing pheochromocytoma whose underlying mechanism has not been elucidated. Herein, we present a case of pheochromocytoma whose clinical manifestations changed, and IL-6 levels elevated over time. A 48-year-old woman was referred to our hospital for fever and hepatic dysfunction. Six years prior, a right adrenal tumor was detected during the examination for ovarian teratoma without C-reactive protein (CRP) elevation. Several imaging studies at our hospital showed no abnormalities except for an increase in the size of the adrenal tumor and hepatomegaly. In addition, antibiotics did not improve the fever. Laboratory tests showed elevated levels of CRP with IL-6 elevation. An enlarged adrenal tumor was detected. Administering doxazosin lowered the CRP and IL-6 levels, then IL-6–producing pheochromocytoma was suspected, and adrenalectomy was performed. After surgery, fever and hepatic function were improved, and the CRP and IL-6 levels were normalized. Immunostaining of the resected tissue showed IL-6 focal positivity, which meant the phenotype of tumor cells focally changed their phenotypes over time. IL-6–producing pheochromocytoma should be considered in patients with adrenal tumors and fever of unknown origin.

## Introduction

Pheochromocytoma is a tumor derived from chromaffin cells in the adrenal medulla, which leads to excessive catecholamine production. Pheochromocytoma is a rare disease, accounting for approximately 0.05% to 0.1% of sustained hypertension [1]. Typical symptoms of pheochromocytoma are palpitation, sweating, headache, hypertension, and pallor due to the excess release of catecholamines [1]. Some pheochromocytomas secrete other bioactive neuropeptides or hormones, including interleukin-6 (IL-6), a cytokine crucial to inflammation and immunity, causing various unusual clinical manifestations [2‐5]. In addition to the typical symptoms of pheochromocytoma, IL-6–producing pheochromocytoma often causes fever and inflammatory syndrome, including hepatic dysfunction, anemia, leukocytosis, and thrombocytosis [2, 3].

To date, approximately 40 cases of IL-6–producing pheochromocytoma have been reported in the literature [2]. Like in typical pheochromocytoma, adrenalectomy for IL-6–producing pheochromocytoma is expected to provide a positive outcome, and its curative effect is reported in the previous literature [2]. In addition, studies have reported that administering either α-blockers or nonsteroidal anti-inflammatory drugs (NSAIDs) suppresses IL-6 production and relieves the clinical manifestations [2].

Herein, we report a case of IL-6–producing pheochromocytoma in which the tumor size and inflammatory symptoms were increased over time.

## Case Presentation

**Figure 1.**
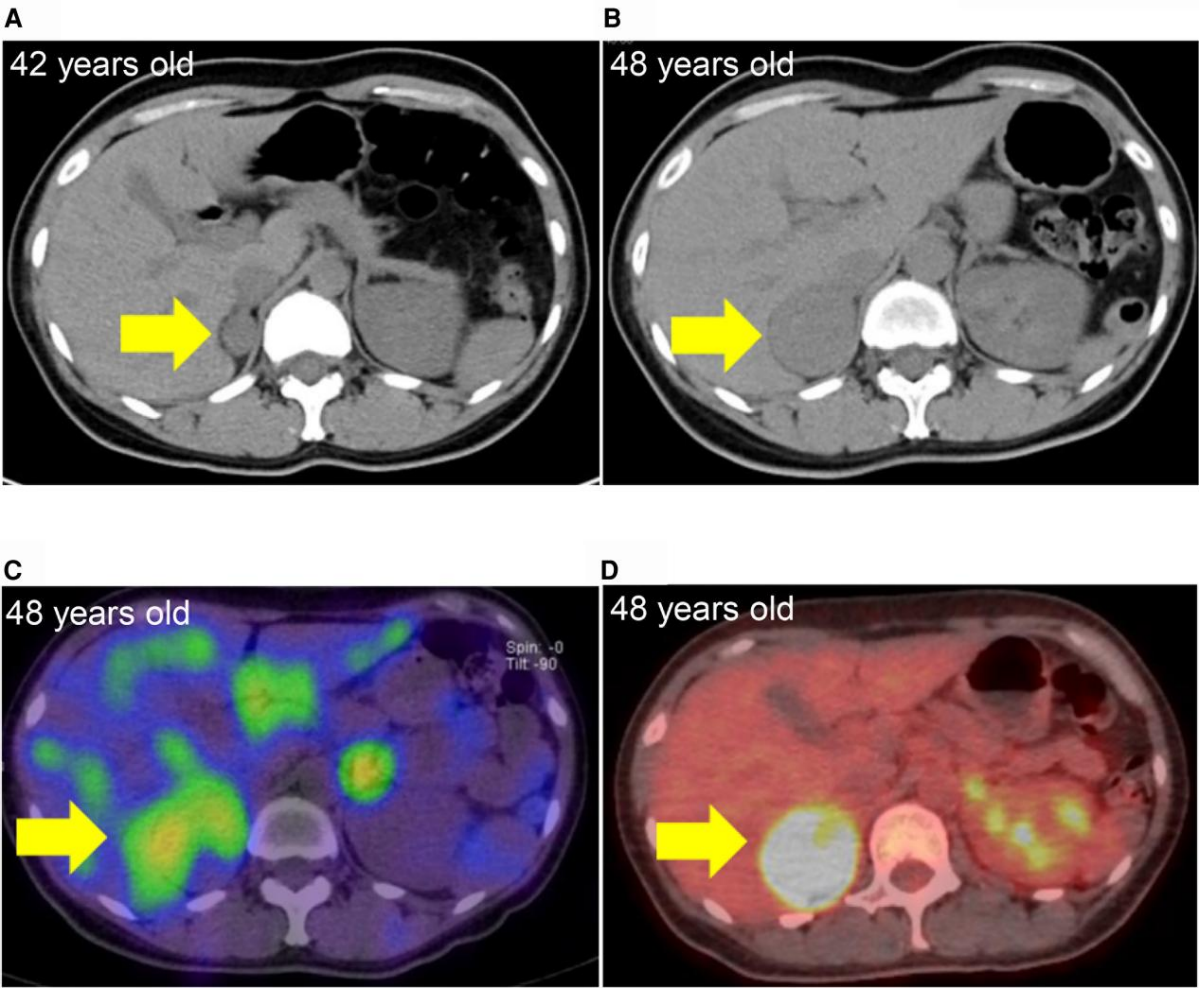
Image findings of the right adrenal gland. A, Computed tomography (CT) image at age 42 years. The arrow indicates a 22-mm tumor. The CT value of the tumor was 36 HU. B, CT image at age 48 years. The arrow indicates a 42-mm tumor. The CT value of the tumor was 43 HU (C) ^123^I-MIBG scintigraphy at age 48 years. The arrow indicates a tumor in the right adrenal gland. D, FDG-PET at age 48 years. The arrow indicates a tumor in the right adrenal gland.

A 48-year-old woman was referred to our hospital with a suspicion of pheochromocytoma. When the patient was aged 42 years, computed tomography (CT) images revealed a tumor at the right adrenal gland with a diameter of 22 mm (Fig. 1) during the examination for ovarian teratoma. However, no symptoms relevant to hormone excess were observed, including hypertension. No laboratory tests suggested the presence of inflammatory markers before the surgical treatment of ovarian teratoma (Table 1). At age 46 years, the patient presented with dizziness and sweating. When she was aged 47 years, a medical examination revealed that fasting blood glucose was 126 mg/dL and glycated hemoglobin A_1c_ was 6.1%.

**Table 1.** Laboratory data 1

	At 42 y (before surgery)	At 48 y (before adrenalectomy)	At 48 y (after adrenalectomy)	Normal value
ACTH, pg/mL	—	5.9	—	<46
Cortisol, nmol/L	—	314.5	—	171.0-535.2
AST, U/L	23	75	24	13-33
ALT, U/L	24	196	40	6-27
γ-GTP, U/L	15	607	—	10-47
CRP, mg/dL	0.02	25.77	0.05	<0.14
IL-6, pg/mL	—	267	1.8	<7
WBC, 10^3^/μL	4.61	7.95	—	3.3-8.8
RBC, 10^6^/μL	4.52	2.87	—	3.7-4.9
Hb, g/dL	12.4	8.2	—	11.2-14.5
Plt, 10^4^/μL	22.1	59.1	—	130-350

Abbreviations: γ-GTP, γ-glutamyl transpeptidase; ACTH, adrenocorticotropin; ALT, alkaline phosphatase; AST, aspartate transaminase; CRP, C-reactive protein; Hb, hemoglobin; IL-6, interleukin-6; Plt, platelet; RBC, red blood cells; WBC, white blood cells.

At age 48 years, the patient presented again with persistent fever, inflammatory responses, and impaired hepatic function and was admitted for treatment at her previous hospital. The patient complained of palpitation and chest oppression; her systolic blood pressure was 200 mm Hg. An endocrinological assessment revealed an elevated level of C-reactive protein (CRP) and IL-6. Therefore, infectious disease was initially suspected.

However, administering medication, including tazobactam/piperacillin and acetaminophen, did not improve fever and paroxysmal hypertension. Another potential cause of the patient's symptoms was an increased size of the right adrenal tumor to 42 mm. Subsequently, the patient was transferred to a tertiary medical facility to examine and treat the adrenal tumor.

## Diagnostic Assessment

The patient's grandfather and grandmother had dyslipidemia and diabetes mellitus, respectively. However, no familial history relevant to the patient's adrenal disease was found. The patient had no history of smoking or alcohol intake. She was taking 5 mg of atorvastatin at the time of admission. On admission, the patient's height, weight, body mass index, blood pressure, pulse rate, and body temperature were 149.5 cm, 47.8 kg, 21.4, 141/94 mm Hg, 106/min, and 37.6 °C, respectively.

CT revealed a 42-mm tumor at the right adrenal gland (see Fig. 1). Fluorodeoxyglucose–positron emission tomography (FDG-PET) showed positive uptake of FDG by the right adrenal tumor. However, metastasis was not observed in the imaging findings.

On physical observation, signs of Cushing syndrome were not noticed. Laboratory tests revealed high levels of liver/biliary enzymes (aspartate transaminase: 75 U/L, alkaline phosphatase: 196 U/L, γ-glutamyl transpeptidase: 607 U/L) (see Table 1). Elevated levels of additional inflammatory markers such as CRP and IL-6 were observed (see Table 1). Thrombocytosis (see Table 1, platelets: 59.1 × 10^4^/μL) and anemia (see Table 1; hemoglobin: 8.2 g/dL) were confirmed. All tests for infectious or autoimmune diseases showed negative results (Table 2). Endocrinological hormones also showed abnormalities. Urine noradrenaline and normetanephrine levels were 8070 µg/day and 12.0 mg/day, respectively, indicating an increased level of norepinephrine (Table 3).

**Table 2.** Laboratory data 2

	Age 48 y before adrenalectomy	Normal value
HBs Ag	(−)	(−)
HBc AB	(−)	(−)
HCV-Ab	(−)	(−)
HA-IgM	(−)	(−)
HEV-IgA	(−)	(−)
IgG, mg/dL	1522	870-1700
IgA, mg/dL	323	110-440
IgM, mg/dL	79	46-260
Antinuclear antibody	x20	<40
Anti-Mit antibody	(−)	(−)
Anti-smooth muscle antibody	(−)	(−)
RF, IU/mL	4	<15
PR3-ANCA	(−)	(−)
MPO-ANCA	(−)	(−)

(−) refers to a negative test result.

Abbreviations: Anti-Mit antibody, antimitochondria antibody; HA-IgM, hepatitis A–immunoglobulin M; HBc, hepatitis B virus antibody; HBs AG, hepatitis B virus antigen; HCV-ab, hepatitis C virus antibody; HEV-IgA, hepatitis E virus–immunoglobulin A; IgG, immunoglobulin G; IgM, immunoglobulin M; MPO-ANCA, myeloperoxidase-antineutrophil cytoplasmic antibody; PR3-ANCA, proteinase 3-antineutrophil cytoplasmic antibody; RF, rheumatoid factor.

**Table 3.** Laboratory data 3

	At 48 y (before adrenalectomy)	At 48 y (after adrenalectomy)	Normal value
Urine adrenaline, μg/d	87.8	8.9	1-23
Urine noradrenaline, μg/d	8070	160.0	29-120
Urine dopamine, μg/d	2600	730	100-1000
Urine metanephrine, mg/d	0.46	0.09	0.05-0.20
Urine normetanephrine, mg/d	12.0	0.28	0.10-0.28

Further, ^123^I-metaiodobenzylguanidline (^123^I-MIBG) scintigraphy was conducted to confirm the presence of pheochromocytoma. The image showed partially positive uptake at the right adrenal tumor (Fig. 1C and 1D).

During the admission, administering doxazosin and naproxen improved inflammatory markers, including CRP, liver/biliary enzymes, and IL-6. The ^123^I-MIBG scintigraphy confirmed pheochromocytoma, and decreases in IL-6 and CRP levels by the α-adrenergic blocker (doxazosin) and NSAID (naproxen) made us suspect IL-6 pheochromocytoma.

## Treatment

Adrenalectomy was performed under 16 mg of doxazosin and 900 mg of naproxen.

## Outcome and Follow-up

**Figure 2.**
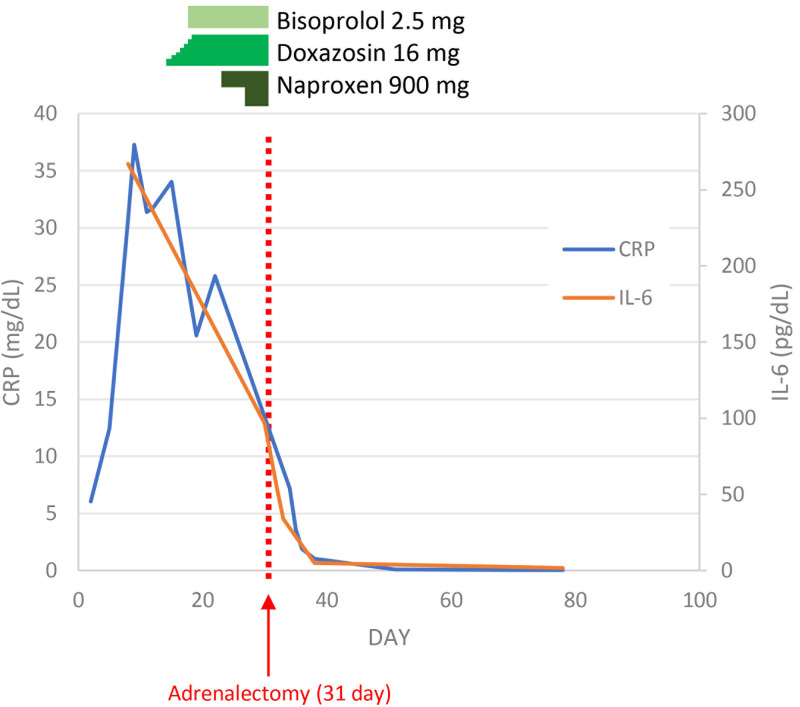
Interleukin-6 (IL-6) and C-reactive protein (CRP) levels before and after treatment.

**Figure 3.**
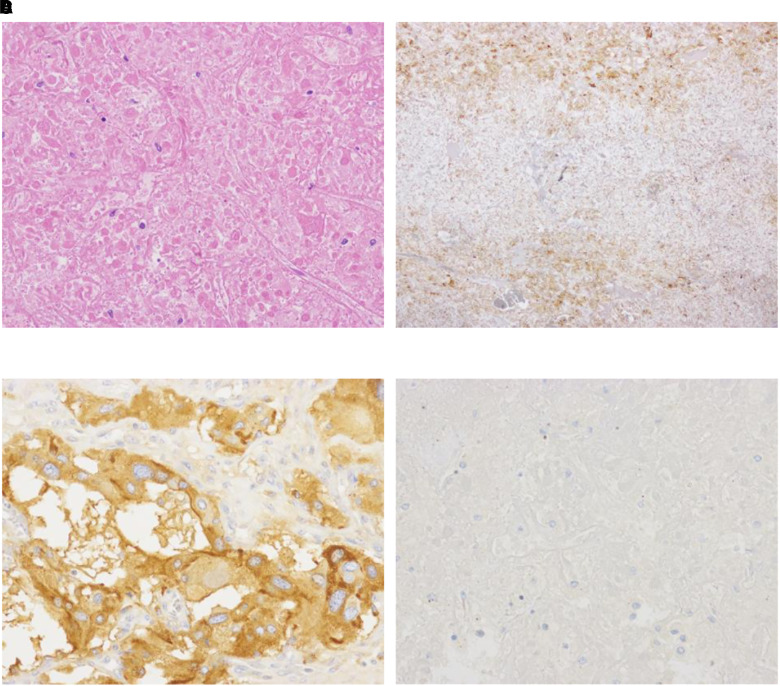
Immunostaining images of the tumor specimen. A, Hematoxylin-eosin staining of the resected tissue. B, Interleukin-6 immunostaining of the resected tissue. Immunostaining showed focal positivity in the resected tissue. C, Chromogranin immunostaining of the resected tissue. Immunostaining showed positive results in the resected tissue. D, Succinate dehydrogenase B immunostaining of the resected tissue. Immunostaining showed negative results in the resected tissue.

**Figure 4.**
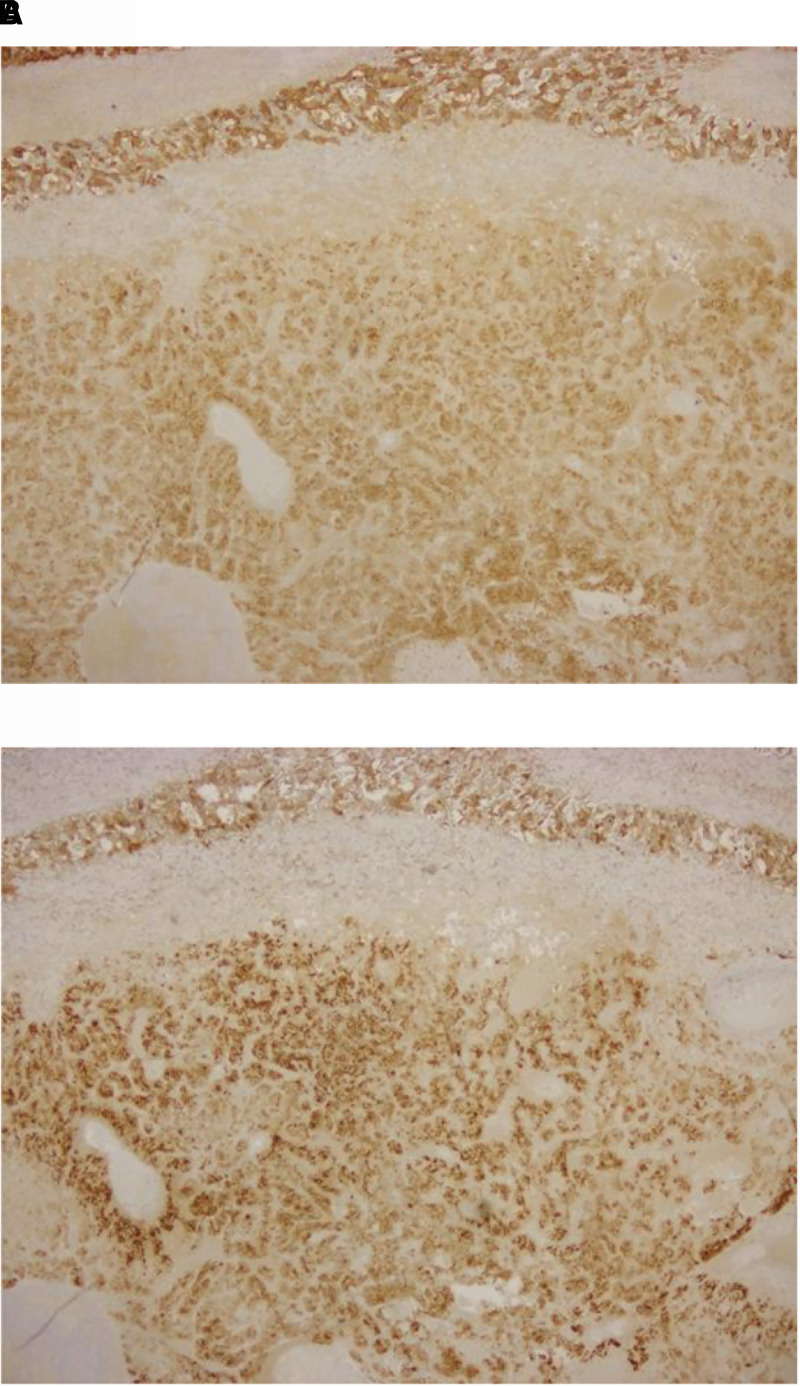
Immunostaining of the tumor specimen (chromogranin and interleukin-6 [IL-6]). Immunostaining of the tumor specimen. Immunostaining images A and B are stained in the same location. A, Chromogranin immunostaining of the resected tissue. Immunostaining showed positive results in the resected tissue. B, IL-6 immunostaining of the resected tissue. Immunostaining showed focal positivity in the resected tissue.

Soon after the surgery, a decrease in catecholamine levels and inflammatory markers was confirmed (see Tables 1 and 3; Fig. 2). Histopathological/genetic analysis of the resected tumor tissue was performed after adrenalectomy. Hematoxylin-eosin stain and chromogranin staining findings were consistent with pheochromocytoma (Figs. 3 and 4). IL-6 immunostaining showed focal positivity, and localization of positivity corresponded to chromogranin (see Figs. 3 and 4). However, succinate dehydrogenase B (SDHB) immunostaining showed a negative test result (see Fig. 3).

In addition, comprehensive gene analysis for typical driver genes of pheochromocytoma was retrospectively investigated. The assessed genes were *RET*, *MEN1*, *NF1*, and *VHL*. Gene analysis revealed the somatic mutation in *VHL*. To better understand the mechanism underlying IL-6 overproduction, immunostaining of Lin 28 was performed; the results were negative.

## Discussion

Our case is the first to report IL-6–producing pheochromocytoma with changes in its clinical characteristics over time. Initially, the patient showed no unusual symptoms, such as fever or elevated inflammatory markers, but she presented with a right adrenal tumor. After a few years, the patient developed symptoms typical of IL-6–producing pheochromocytomas, such as fever and hepatic dysfunction. This might suggest a possibility of acquired IL-6–production ability with pheochromocytoma.

Pharmacological strategies to suppress the symptoms of IL-6–producing pheochromocytoma can be NSAIDs or α-blockers [2, 3]. Reports from the literature revealed that NSAIDs and α-blockers reduced IL-6 or CRP levels and improved its symptoms. However, some cases did not show improvement after administering these drugs [2]. The mechanism of IL-6 pheochromocytoma remission under NSAID or α-blockers remains unclear. Meijs et al [2] suggest that naproxen interferes with the posttranslational modification of IL-6 protein, which may be attributed to NSAID effectiveness. They also claim that α-blockers suppress the production of tumor necrosis factor-α, which may reduce the IL-6 or CRP level. In the present case, administering doxazosin reduced the IL-6 level. Based on the previous reports and our case, the optimal medication for patients with IL-6 pheochromocytoma depends on each case. Thus, the prescription should be carefully considered.

The pathogenesis of IL-6 overproduction with pheochromocytoma is discussed in the literature [2, 4]. The 2 major hypotheses explaining this are that IL-6 is directly produced by the tumor or indirectly produced attributed to the tumor [4]. Yarman et al [4] argued that excess production of norepinephrine from the tumor leads to a high level of IL-6. However, IL-6–producing pheochromocytoma with a normal level of epinephrine was previously reported, which may imply a direct production of IL-6 from the pheochromocytoma [5].

Various genetic mutations have been reported in pheochromocytoma, but their association with IL-6 is not fully elucidated. This is because most previously reported patients with IL-6–producing pheochromocytoma either had no mutation or were not screened for gene mutations [2]. In our case, the SDHB immunostaining results suggested that the tumor may have an *SDHB* gene mutation. In addition, comprehensive gene analysis revealed a somatic mutation at *VHL* from the pheochromocytoma. VHL protein plays a role in hypoxia-inducible factor-1α degradation and suppresses vascular endothelial growth factor transcription and tumor progression [6].

Therefore, the loss of function of the VHL protein causes angiogenesis and tumor progression. Considering the acquisition of IL-6 secretion, we hypothesized that IL-6 secretion directly from the tumor may be attributed to Lin 28, a stem cell marker of RNA binding protein. Previous studies on lung cancer demonstrated that Src activation triggers Lin28 production, which gives the tumor the ability to produce IL-6 [7, 8]. Lin28 inhibits let-7, a microRNA, which directly inhibits IL-6 expression post transcriptionally, and enables the tumor to produce IL-6 [7, 8]. Recently, studies revealed that Src activation also degrades VHL proteins [6]. Production of VHL oncoprotein could have activated the Src–Lin 28 pathway to degrade VHL oncoproteins. However, pheochromocytoma tissue was negative for Lin 28 immunostaining in the present case. Although *VHL* somatic mutation could have been involved in tumor progression, other pathological mechanisms may exist.

Other than pheochromocytoma, tumor-infiltrating macrophages could be associated with IL-6 production. A recent study has revealed the presence of intratumor macrophage in the pheochromocytoma tissue [9]. The tumor-infiltrating macrophage may be responsible for the IL-6 production in IL-6–producing pheochromocytoma. However, immunohistopathological analysis in the present case showed chromogranin and IL-6 foci–positive results with the same localization, implying that IL-6 production came directly from the pheochromocytoma cells.

The fact that tumor growth and excess IL-6 production occurred over time in the present case with incidentaloma emphasizes the importance of careful examination by endocrinologists in patients with adrenal incidentalomas or nonfunctional adrenal tumors. Spiro et al [10] reported a case that helps us to understand further the nonfunctional pheochromocytoma. They presented a case with pheochromocytoma found as incidentaloma. The biochemical evaluation showed no abnormalities for the first 3 years, and the patient was asymptomatic. With time, the elevation of urine normetanephrine and progression of the adrenal tumor were confirmed. These cases may indicate that pheochromocytoma can initially be nonfunctional and change its biochemical condition or symptoms through tumor growth. Following-up patients with incidentaloma that is nonfunctional or symptomatically silent, through imaging modalities and biochemical evaluations, is recommended.

IL-6–producing pheochromocytoma has various clinical manifestations that prevent making a diagnosis [2]. In the present case, unusual symptoms included hepatic dysfunction, thrombocytosis, and anemia. Furthermore, previous studies reported the presence of leukocytosis in some IL-6–producing pheochromocytoma [2, 3]. Excess IL-6 may injure liver cells and lead to hepatic dysfunction [2]. IL-6 can also induce B-cell differentiation and thrombopoiesis, which may be involved in leukocytosis and thrombocytosis [2]. Fever and elevated IL-6 levels might be the key symptom and an important biochemical marker of IL-6 pheochromocytoma. Cheng et al [5] demonstrated a positive correlation between pheochromocytoma with body temperature and IL-6 level in the pheochromocytoma tissue. However, some cases without pyrexia were previously reported in the literature, which implies that an elevated IL-6 level does not always induce fever [2]. Further exploration is needed to determine the clinical features and biochemical markers of IL-6–producing pheochromocytoma.

In conclusion, clinical characteristics of pheochromocytoma might change over time, causing IL-6 secretion, which could present with numerous atypical symptoms and thus hinder the diagnosis. If a patient presents with an adrenal tumor along with fever or high IL-6 levels, clinicians should also consider IL-6–producing pheochromocytoma during the diagnosis. We recommend an early examination of the adrenal tumor after its detection to avoid a potential misdiagnosis.

## Learning Points

- The phenotype of the adrenal tumor cells might change over time, causing additional unusual clinical characteristics such as IL-6 overproduction and fever with an increase in the size of the adrenal tumor.
- Clinicians should consider IL-6–producing pheochromocytoma when a patient presents with an adrenal tumor with elevated inflammatory responses and fever.
- Even if an adrenal tumor seems nonfunctional, an early examination of the tumor by endocrinologists is recommended.

## Data Availability

Data sharing does not apply to this article as no data sets were generated or analyzed during the current study.

## Abbreviations

^123^I-MIBG: ^123^I-metaiodobenzylguanidline
CRP: C-reactive protein
CT: computed tomography
FDG-PET: fluorodeoxyglucose–positron emission tomography
IL-6: interleukin-6
NSAID: nonsteroidal anti-inflammatory drug
SDHB: succinate dehydrogenase B
